# Sphenopalatine Ganglion Block for the Treatment of Acute Migraine Headache

**DOI:** 10.1155/2018/2516953

**Published:** 2018-05-07

**Authors:** Mohamed Binfalah, Eman Alghawi, Eslam Shosha, Ali Alhilly, Moiz Bakhiet

**Affiliations:** ^1^University Medical Center, King Abdullah Medical City, P.O. Box 26671, Adliya, Bahrain; ^2^Ministry of Health, Building 1228, Road 4025, 340 Juffair, Bahrain; ^3^Majmaah University, Academic City, Al Majmaah 15341, Saudi Arabia; ^4^Bahrain Defense Force Hospital, P.O. Box 28743, West Riffa, Bahrain; ^5^Aljawhara Center, Arabian Gulf University, P.O. Box 26671, Manama, Bahrain

## Abstract

Transnasal sphenopalatine ganglion block is emerging as is an attractive and effective treatment modality for acute migraine headaches, cluster headache, trigeminal neuralgia, and several other conditions. We assessed the efficacy and safety of this treatment using the Sphenocath® device. 55 patients with acute migraine headaches underwent this procedure, receiving 2 ml of 2% lidocaine in each nostril. Pain numeric rating scale (baseline, 15 minutes, 2 hours, and 24 hours) and patient global impression of change (2 hours and 24 hours after treatment) were recorded. The majority of patients became headache-free at 15 minutes, 2 hours, and 24 hours after procedure (70.9%, 78.2%, and 70.4%, resp.). The rate of headache relief (50% or more reduction in headache intensity) was 27.3% at 15 minutes, 20% at 2 hours, and 22.2% at 24 hours. The mean pain numeric rating scale decreased significantly at 15 minutes, 2 hours, and 24 hours, respectively. Most patients rated the results as very good or good. The procedure was well-tolerated with few adverse events. This treatment is emerging as an effective and safe option for management of acute migraine attacks.

## 1. Introduction

Migraine is a common primary headache disorder, causing significant disability and personal, societal, and financial burden [1]. It is a highly prevalent condition, affecting 11% of adult population worldwide, including people of all ages, races, geographical areas, and income levels [2]. Although there are currently many options for acute migraine treatment, such as acetaminophen, nonsteroidal anti-inflammatory drugs (NSAIDS), triptans, combinations analgesics, and antiemetics [3], these treatment options are often suboptimal, with inadequate efficacy and significant side effects [4, 5]. In addition, several studies [6–8] have shown that migraine patients with poor response to acute treatment are at increased risk for transformation to chronic migraine (CM), with roughly 2.5-3.5-fold greater odds of developing CM [6]; patients with a moderate or better acute treatment efficacy did not have a significant increased risk. Therefore, there is a continuous need for new treatment modalities to address the therapeutic needs of migraine sufferers, especially those with frequent and disabling attacks [9].

**Figure 1:**
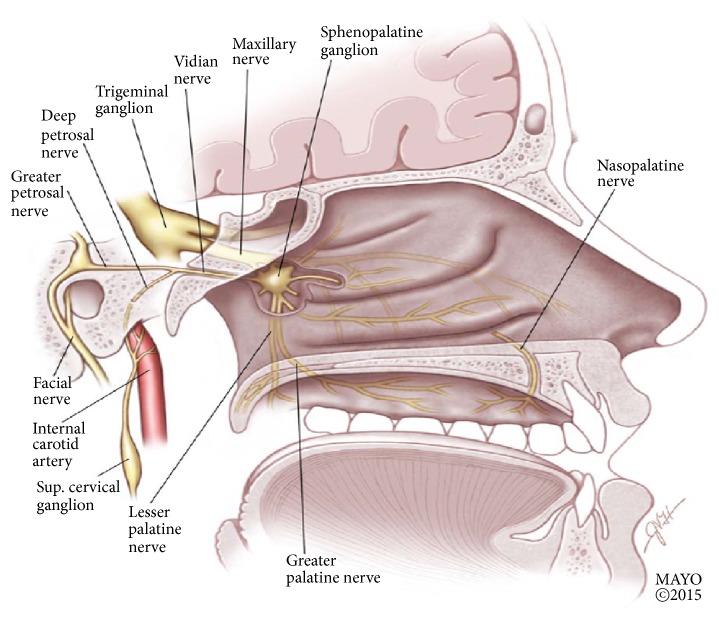
Saggital view of the nasopharynx, showing the sphenopalatine ganglion and its neural connections. Reproduced with permission from Robbins et al. (2016) [under the Creative Commons Attribution License number 4318850197898 (Wiley).

Sphenopalatine ganglion (SPG) block has gained interest as an effective treatment modality for migraine and other headache and facial pain syndromes [10]. SPG, also known as the pterygopalatine ganglion (PPG), is a large extracranial parasympathetic ganglion with multiple neural connections (Figure 1), including autonomic, motor, and sensory [11, 12]. This complex neural structure is located deeply in the pterygopalatine fossa (PPF) posterior to the middle turbinate and maxillary sinus [11], on each side of the face. The parasympathetic preganglionic cell bodies originate in the superior salivatory nucleus in the pons, and the parasympathetic fibers run in the nervus intermedius (a branch from the facial nerve) through the geniculate ganglion, forming the greater petrosal nerve (GPN). The sympathetic fibers originate in the superior cervical ganglion around the internal carotid artery and give rise to the deep petrosal nerve, which joins the GPN to form the Vidian nerve, which enters the SPG. The sensory input to the SPG is via branches from the maxillary nerve, carrying sensations from the palate, buccal cavity, gingival, and tonsils [10].

The parasympathetic fibers synapse in the SPG and second-order neurons provide secretomotor function to the mucous membranes of nose, mouth, pharynx, and lacrimal glands, as well as branches to the meningeal and cerebral blood vessels [10, 12, 13]. The sympathetic fibers pass through the SPG without synapsing and provide innervations to the palate, nasal cavity, and pharynx.

As acute migraine attacks, as well as other primary headache disorders like cluster headache, are often associated with signs of parasympathetic activation, including lacrimation, nasal congestion, and conjunctival injection, blocking the SPG, which is the major parasympathetic outflow to the cranial and facial structures, is a reasonable target to help relief pain and autonomic features seen in these disorders [14]. It is proposed that various migraine triggers activate brain areas related to superior salivatory nucleus, leading to stimulation of the trigemino-autonomic reflex. This results in increased parasympathetic outflow from the SPG, causing vasodilatation of cranial blood vessels that happens during migraine [10, 14], with the release of inflammatory mediators from blood vessels and activation of meningeal nociceptors, causing migraine pain [11, 14]. Another possible effect of SPG block is modulation of sensory processes in the trigeminal nucleus caudalis via the afferent sensory fibers, which may change pain processing center and reduce central sensitization to pain that is commonly seen in migraine [9, 10].

SPG blocks have been used for the treatment of headache since a long time [10]. In 1908, Sluder described the use of transnasal SPG block using a long needle to inject cocaine, treating what was called Sluder's neuralgia [15]. The technique was further developed by Simon Ruskin [16], and in 1925 he used it to treat trigeminal neuralgia. Since then, the indications for SPG block have expanded to include cluster headache, migraine, trigeminal neuralgia, and many more [10, 17–19].

SPG blocks have been achieved with various techniques, including the use of lidocaine-soaked cotton tip applicator through the nose, transorally, transnasal endoscopic, infratemporal approach, and more recently using various noninvasive transnasal devices to inject anesthetics into the SPG [19].

The objective of this study is to assess the efficacy of SPG block, using the Sphenocath device, for the treatment of acute migraine headaches in the outpatient setting. We also report the safety of this novel technique for migraine treatment.

## 2. Methods

### 2.1. Study Design and Setting

We conducted an open, uncontrolled, retrospective study in the neurology clinic at a university medical center. The patients were treated between March 2017 and September 2017. The study was approved by the institutional review board of University Medical Center at King Abdullah Medical City.

### 2.2. Study Population

The patients were recruited to the study if they were between 18 and 60 years of age, have been diagnosed with migraine headache according to International Classification of Headache Disorders-3 Beta [20] since at least one year, and present with moderate to severe headache lasting between 4 and 72 hours not responding to abortive medications. Patients with medication overuse headache, bleeding disorders, abnormal neurological examination, and history of allergy to local anesthetics were not included in the study. All patients gave an informed written consent.

### 2.3. Methods of Measurement

Pain was assessed using numeric rating scale (NRS), where 0 is no pain and 10 is worst pain imaginable; this was recorded at baseline, 15 minutes, 2 hours, and 24 hours after the procedure. We also recorded patient global impression of change (PGIC; very poor, poor, no change, good, and very good) at 2 hours and 24 hours after procedure.

### 2.4. Outcome Measures

The primary efficacy measure was the percentage of patients free of headache at 15 minutes, 2 hours, and 24 hours after the procedure. Secondary endpoints were

- headache relief rate, defined as percentage of patients with 50% or more reduction in headache intensity at 15 minutes, 2 hours, and 24 hours;
- change in NRS from baseline to 15 minutes, 2 hours, and 24 hours after treatment;
- PGIC (effects on headache and its associated symptoms and tolerability) at 2 hours and 24 hours;
- all adverse events up to 24 hours after procedure.

Statistical analysis was done using SPSS Statistics Version 23.

## 3. Procedure

**Figure 2:**
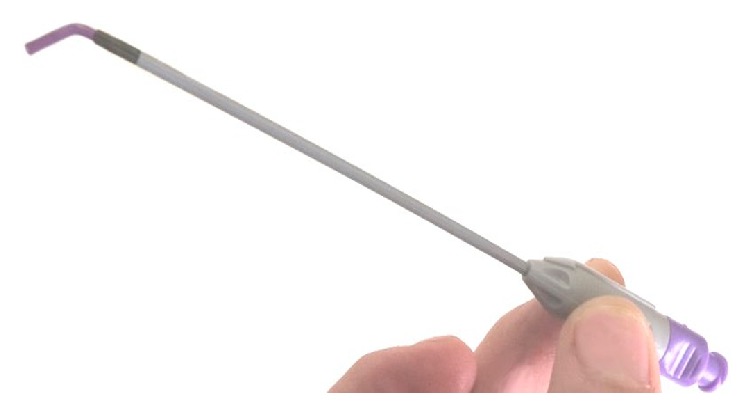
The Sphenocath device. Image provided courtesy of Dolor Technologies.

Prior to procedure, the nose was inspected for any obstruction, and xylometazoline 0.05% nasal drops (one drop in each nostril) were used to help open the nasal passages. Face temperature was recorded using temperature sensor skin probes put on both cheeks. A small amount of 2% lidocaine jelly was installed in each nostril for patients' comfort, using a needless syringe. Each patient received a single treatment of transnasal SPG block with 2 cc of 2% lidocaine in each nostril in the supine position with head extension, delivered using the Sphenocath device. This is a small flexible sheath with a curved tip (Figure 2). It is inserted through the anterior nasal passage parallel to nasal septum and above the middle turbinate. Once in place, the inner catheter is advanced to administer 2 cc of 2% lidocaine. It is then removed and the procedure is repeated on the other side. Typically after the block, there is an increase in face temperature by 1 to 2 degrees Celsius and/or tearing [21]. The patient is instructed to remain in the same position for 10 minutes.

## 4. Results

**Figure 3:**
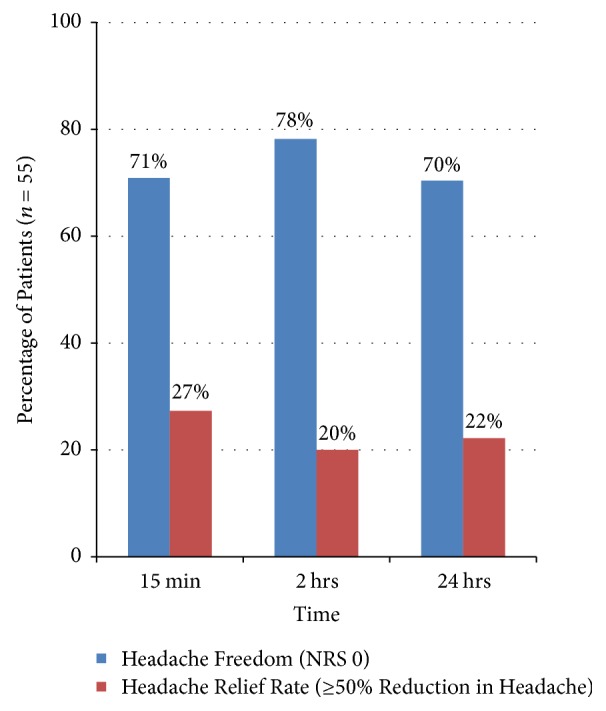
The percentage of patients reaching headache freedom (pain numeric rating scale 0) and patients with headache relief (50% or more reduction in headache intensity), at 15 minutes, 2 hours, and 24 hours.

55 patients received treatment with bilateral transnasal SPG blocks. 72.7% were females. The age range of patients was 19 to 58 years, with a mean age of 37.9 years. The baseline NRS range was 4 to 10, with a mean of 6.8. For the primary end point (headache freedom at 15 minutes, 2 hours, and 24 hours), the percentages were 70.9%, 78.2%, and 70.4%, respectively (Figure 3). Among the secondary efficacy measures, 27.3%, 20%, and 22.2% of patients reported headache relief at 15 minutes, 2 hours, and 24 hours after the procedure, respectively (Figure 3).

**Figure 4:**
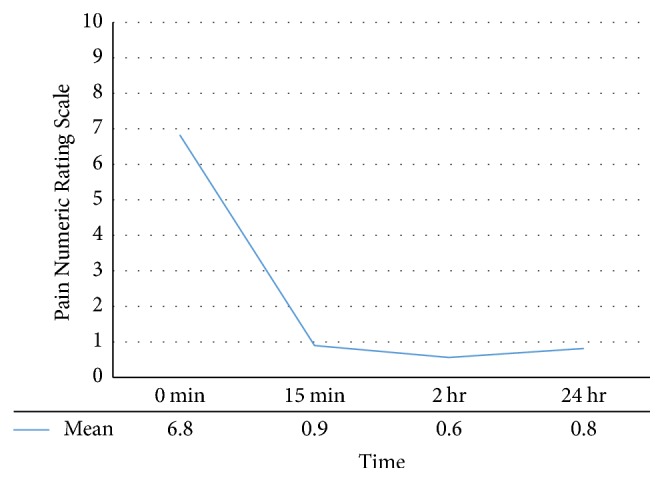
The mean pain numeric rating scale at baseline and 15 minutes, 2 hours, and 24 hours after treatment, showing significant and sustained reduction in pain intensity.

The mean NRS scores decreased significantly from a baseline of 6.8 to 0.9, 0.6, and 0.8 at 15 minutes, 2 hours, and 24 hours after procedure, respectively (Figure 4).

**Figure 5:**
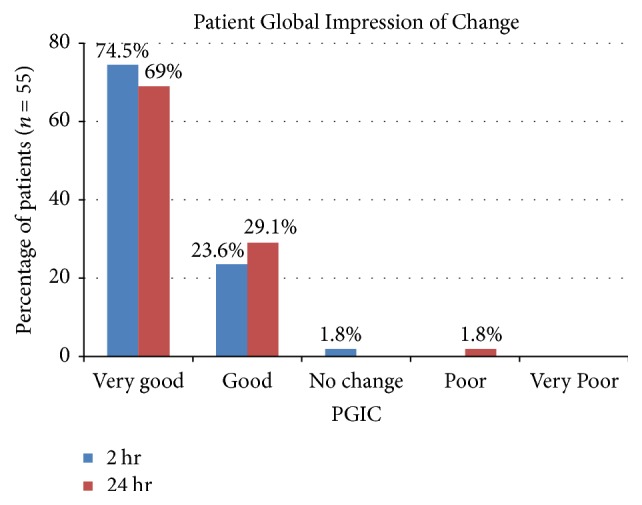
Patient global impression of change after the procedure at 2 hours and 24 hours. The majority of patients rated the treatment result as very good or good.

Regarding PGIC, the majority of patients (98.1% at 2 hours, 98.1% at 24 hours) reported feeling very good or good (Figure 5). Only one patient reported “no change” in PGIC scale at 2 hours, but “very good” at 24 hours, and another patient rated her PGIC as “good” at 2 hours and “poor” at 24 hours due to return of headache which was slightly worse than before.

**Figure 6:**
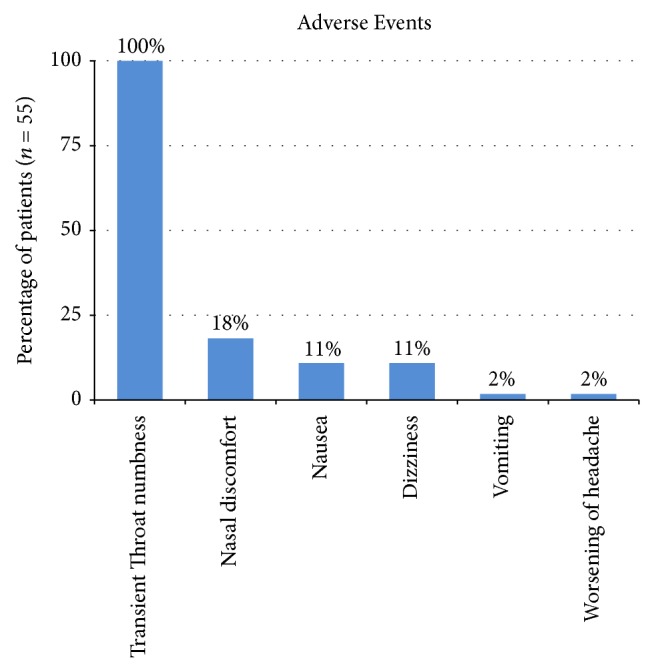
Adverse events recorded in the first 24 hours after the procedure.

Overall, the procedure was well-tolerated. Adverse events reported by the study population were mild (Figure 6), including transient throat numbness (100%), nausea (10.9%), dizziness (10.9%), vomiting (1.8%), nasal discomfort (18.2%), and worsening of preexisting headache (1.8%). These adverse events were transient and lasted less than 24 hours.

## 5. Discussion

This retrospective case series demonstrated that transnasal SPG block with 2% lidocaine, using the Sphenocath device, is an effective and safe treatment for acute migraine headaches. There was a rapid relief of headaches observed at 15 minutes and 2 hours, and treatment effect was sustained at 24 hours after procedure in most patients. 70.9%, 78.2%, and 70.9% of patients were completely headache-free at 15 minutes, 2 hours, and 24 hours, respectively, while further 27%, 20%, and 27% achieved 50% or more headache relief at 15 minutes, 2 hours, and 24 hours, respectively. The majority of study population reported either very good or good response on PGIC at 2 hours and 24 hours.

A number of studies were published over the years regarding SPG blockade in acute migraine, with variable results [10]. Kudrow et al. [22] conducted a noncontrolled study in migraine patients using 4% intranasal lidocaine and showed that 12 out of 23 patients achieved complete headache relief, and the effect was sustained at 24 hours. Maizels and Geiger [23] evaluated the efficacy of 4% intranasal lidocaine as a treatment for acute migraine attacks, which was administered by the patient at home, in a double-blind, randomized controlled study. There was a significant reduction in headache severity at 15 minutes compared to placebo, but there was headache recurrence in 21% of patients receiving lidocaine.

Another placebo-controlled study compared outcomes for acute treatment of chronic migraine patients with intranasal 0.5% bupivacaine (*n* = 26) or saline (*n* = 12) using the Tx 360® device to block the SPG [24]. The injection was given twice a week for 6 weeks. The trial revealed significant reduction in pain numeric rating scores in the bupivacaine group at 15 minutes, 30 minutes, and 24 hours after each treatment. A randomized, double-blind, placebo-controlled study using intranasal bupivacaine or saline injections in patients presenting to the emergency department with acute frontal-based headache [specific classification was not required] demonstrated no significant difference in the proportion of patients achieving 50% or more headache relief at 15 minutes [25].

Other studies used different agents for SPG blockade. For example, Bratbak et al. used onabotulinum toxin A injections into the SPG in 10 patients with intractable chronic migraine in an open, uncontrolled study [26]. This was done through a percutaneous infrazygomatic approach with a novel injection device. A statistically significant reduction of moderate and severe headaches was observed at 2 months after treatment; there were a total of 25 adverse events, mostly local discomfort, but none were classified as severe.

The SPG unique position in the PPF, as well as its multiple neural connections to sensory and autonomic systems involved in pain generation and propagation and the associated autonomic manifestations seen in many primary headache and facial pain syndromes, makes it a promising target for the treatment of these conditions. Inhibition of parasympathetic outflow from the SPG causes reduced activation of perivascular pain receptors in the cranial and meningeal blood vessels, with resultant reduction in the release of neuroinflammatory mediators (acetylcholine, nitric oxide, vasoactive intestinal peptide, substance P, and calcitonin gene-related peptide) from sensory fibers supplying the cranial and meningeal vasculature. This, in turn, reduces pain intensity and intracranial hypersensitivity observed in migraine [14].

In our study, SPG blockade produced a rapid relief of headache at 15 minutes, with a significant treatment effect observed at 24 hours and high patient satisfaction. In general, the treatment was well-tolerated. We recorded few adverse events, which were mild and transient, similar to those seen in previous studies [24].

The main limitation of our study included the lack of a placebo group, as subjective pain response might have a significant placebo component [27]. However, the high treatment response and satisfaction rates in this study were both encouraging and clinically meaningful for our patients. We did not assess the use of analgesics after two hours of receiving the SPG block, which might have influenced the headache relief percentage at 24 hours. However, this is allowed in acute headache trials guidelines [28].

## 6. Conclusion

Transnasal SPG blockade is emerging as an effective and safe option for the treatment of several disabling headache and facial pain conditions such as migraine, cluster headache, and trigeminal neuralgia. Its ease of administration using noninvasive devices, safety profile, and quick pain relief makes it an attractive treatment option for these conditions. More well-designed studies are needed to further explore the efficacy of this treatment modality and its use as part of a comprehensive headache management program.
